# Clinical impact of trace elements as potential biomarkers for diagnosis and prediction of response to systemic treatment in gastrointestinal cancers

**DOI:** 10.2478/raon-2026-0023

**Published:** 2026-09-07

**Authors:** Martina Rebersek, Radmila Milacic Scancar, Janez Scancar, Nezka Hribernik

**Affiliations:** 1 Department of Medical Oncology, Institute of Oncology Ljubljana, Ljubljana, Slovenia; 2 Medical Faculty, University of Ljubljana, Ljubljana, Slovenia; 3 Jožef Stefan Institute, Ljubljana, Slovenia; 4 Jožef Stefan International Postgraduate School, Ljubljana, Slovenia

**Keywords:** trace elements, prognostic and predictive biomarkers, gastrointestinal cancers, systemic therapy

## Abstract

**Background:**

Gastrointestinal cancers are among the most common malignancies worldwide and, at advanced stages, remain incurable. Currently used biomarkers, such as tumour markers, have limited diagnostic value for early detection and relapse, highlighting the urgent need for more sensitive and specific markers. Trace elements are involved in numerous physiological and metabolic processes, and deregulation of their homeostasis is implicated in the carcinogenesis of various cancers, including gastrointestinal malignancies. Several basic and preclinical studies have identified the importance of trace elements in key biological processes. Recent clinical studies and retrospective analyses suggest that fluctuations in trace element levels may be associated with the development and progression of many cancers. Thus, quantitative and dynamic determination of serum trace element concentrations during treatment and follow-up represents a promising option for monitoring treatment efficacy and disease prognosis.

**Conclusions:**

Trace elements may serve as potential prognostic and predictive biomarkers for diagnosis and response to systemic treatment.

## Introduction

Gastrointestinal (GI) cancers comprise a heterogeneous group of diseases affecting the gastrointestinal tract, including cancers of the oesophagus, stomach, liver, biliary tract, pancreas, small intestine, colorectal (CRC), and anus. These cancers vary in aetiology and clinical management and are among the most prevalent malignancies globally, representing a leading cause of cancer-related death.^1^ According to the Cancer Registry of the Republic of Slovenia, nearly 3,000 new cases of gastrointestinal cancer are diagnosed annually.^2^ Prognosis has improved significantly over the past decade due to successful screening programmes, advances in surgical and local ablative techniques, radiation therapy, and systemic treatments for both early and advanced disease. In Slovenia, the incidence of CRC has declined in recent years, primarily due to increased awareness and preventive screening.^2^

Despite these advances, cancer remains a leading cause of death worldwide.^1^ Metastatic GI cancers are still incurable for most patients, with prognosis varying by cancer type, location, and extent of metastases. Pancreatic cancer has the poorest prognosis, while metastatic CRC has a better survival rate, largely due to combined surgical and systemic treatments, especially new systemic treatment possibilities.^3,4^

Early diagnosis greatly improves survival and treatment outcomes, making reliable biomarkers essential. Widely accepted biomarkers for GI cancers are still lacking. Currently used serum tumour markers, carbohydrate antigen 19-9 (CA 19-9) and carcinoembryonic antigen (CEA), have limited diagnostic value due to low sensitivity and specificity.^3-5^ CA 19-9 has higher specificity than CEA (92.7% *vs*. 79.2%) but lower sensitivity (50% *vs*. 79.4%).^5^ Elevated CA 19-9 is associated with poorer prognosis and may serve as a predictive biomarker for systemic treatment response, but it is not cancer-specific and can be elevated in benign liver diseases and other metastatic cancers.^3-5^ Serum CEA is also insufficiently sensitive or specific for GI cancer diagnosis and can be elevated in other cancers and non-malignant diseases. Improved understanding and application of traditional tumour biomarkers, alongside identification of new biomarkers, is crucial for personalized cancer treatment.

## Trace elements

Essential trace elements, including iodine (I), copper (Cu), iron (Fe), manganese (Mn), zinc (Zn), selenium (Se), cobalt (Co), and molybdenum (Mo), are required in minute amounts for normal physiology.^6^ Alterations in levels and changes in the expression of proteins involved in trace element metabolism have been demonstrated in various cancers, including GI malignancies.^6-8^ Cu, Zn, and Fe are particularly important for normal bodily function.^6-8^ They participate in numerous biochemical reactions, act as enzyme cofactors, and regulate biological processes by binding to specific receptors and transcription factors. Deregulation of trace metal homeostasis at the cellular and tissue level is implicated in cancer pathology, accelerating the transformation of normal cells into cancerous cells and altering immune responses.^6-8^

Cu is an essential trace element that is tightly regulated in the body.^9^ It is present in all tissues, stored mainly in the liver, and transported in the blood, mostly bound to ceruloplasmin (Cp).^9-11^ It acts as a coenzyme for several enzymes, including Cu/Zn superoxide dismutase, Cp, cytochrome oxidase, tyrosinase, dopamine hydroxylase, lysine oxidase, catalase, and Se-dependent peroxidase, all of which are crucial for cellular respiration and defines against free radicals. Cu also affects glutathione function, and its deficiency impairs cellular respiration and the regulation of reactive oxygen species. Excessive oxidative stress, due to the overproduction of reactive oxygen species, impairs deoxyribonucleic acid (DNA) repair mechanisms and is a key factor in cancer development.^11^

Cu and Zn are essential micronutrients involved in antioxidant functions, immune regulation, and DNA repair. Cu can promote oxidative stress and inflammation, while Zn has antioxidative properties. Imbalances in Cu and Zn disrupt homeostasis, increasing oxidative stress and inflammation, which are implicated in CRC development. Cancer patients often exhibit higher serum Cu and lower Zn levels than healthy individuals.^11-17^ These differences vary with diet, sex, age, cancer type, and other factors. Low Zn and elevated Cu can increase oxidative stress and impair antioxidant enzyme activity.^17^ Increased Cu/Zn ratios have been observed in various malignancies, including GI, gynaecological, breast, and lung cancers, and correlate with disease stage.^11-17^ The Cu/Zn ratio reflects the balance between Cu and Zn, is crucial for regulating oxidative stress and inflammation, and may serve as a clinical diagnostic and prognostic biomarker for treatment response. Fe is a key mineral for survival, as it helps cells in transport of oxygen and to function properly.^6^ It is essential for the activity of enzymes involved in cellular respiration and the conversion of food into energy. Fe helps the body respond to infections and maintain a healthy immune system. It plays a crucial role in brain development, cognitive function, hormone synthesis, and connective tissue health. The body stores Fe in the liver, spleen, and bone marrow in the form of a protein called ferritin. Se is an essential mineral that acts as a powerful antioxidant and is essential for the smooth functioning of several body processes.^6^ It is necessary for the conversion of thyroid hormones into their active form, strengthens the body’s natural resistance and protects cells from oxidative stress. Se also plays a role in fertility and in hair and nail health.

## Methods for determining trace element concentrations in biological samples

The determination of trace elements in biological samples is essential for under-standing their role in human health and disease. Various analytical techniques are used for analysing trace element concentrations in biological matrices, such as blood, tissue, and urine.^18^ Among these, inductively coupled plasma mass spectrometry (ICP-MS) is the most sensitive and versatile.^18^ It enables rapid simultaneous detection of nearly all elements in the periodic table at extremely low concentrations (below 0.01 μg/L). ICP-MS combines an inductively coupled plasma with a mass spectrometer, which identifies ions based on their mass-to-charge ratio (m/z). This technique can also be used for isotope ratio measurements. The advantages of ICP-MS include its ability to perform rapid, simultaneous multi-elemental analysis, high sensitivity and selectivity, and a wide operational dynamic range (up to 10^9^). To ensure accurate determination of elemental concentrations, it is crucial to minimize or eliminate spectral interferences, such as polyatomic and isobaric interferences, as well as nonspectral interferences that arise from compounds in the sample. These interferences can affect transport efficiency and nebulization. Due to its exceptional performance, ICP-MS has become the fastest growing analytical technique for trace element analysis, particularly for elements in biological matrices.^18^

Similarly, to ICP-MS, inductively coupled plasma atomic emission spectrometry (ICP-AES) also uses a high-temperature argon plasma to excite atoms and ions in a sample, which emit elementspecific radiation that is measured for quantitative analysis.^18^ It offers moderate to high sensitivity, typically in the μg/L to low mg/L range, making it suitable for routine multi-element determination. Although it is less sensitive than ICP-MS, it can also be used for trace element determination in biological matrices. The method is primarily affected by spectral interferences from overlapping emission lines and by matrix effects that can influence signal intensity. However, careful selection of alternative emission lines and mathematical correction techniques can minimize these interferences.

Flame and electrothermal atomic absorption spectrometry (FAAS and ETAAS) are much less sensitive than ICP-MS, with typical detection limits in the mg/L range for FAAS and μg/L range for ETAAS, and they allow the determination of only a single element per analysis.^18^ For this reason, they are less commonly used for trace element analysis in biological samples compared with ICP-MS. The technique is based on the ability of atoms in the atomization medium (flame or electrothermally heated graphite furnace) to absorb the characteristic light of the element. The most common interferences are chemical and spectral interferences, as well as background non-specific absorption caused by scattering or absorption from other matrix components. These effects can be minimized by using matrix modifiers, optimized furnace programs, or background correction techniques such as continuum source or Zeeman correction.^18^

Total Reflection X-ray Fluorescence (TXRF) enables direct analysis of liquids, slurries, and solid tissues with minimal sample preparation, providing moderate sensitivity in the μg/L to mg/L range, which makes it suitable for trace element determination in biological samples.^18^

Neutron activation analysis (NAA) is a highly sensitive, matrix-independent technique, often achieving ng/L to μg/L detection limits, and offers absolute quantification, making it particularly valuable for trace element analysis in biological ma-trices.^18^ However, it is time-consuming and requires access to a nuclear reactor, which, for most of the laboratories, is not available.

## Trace elements as potential biomarkers in oncology

**FIGURE 1.**
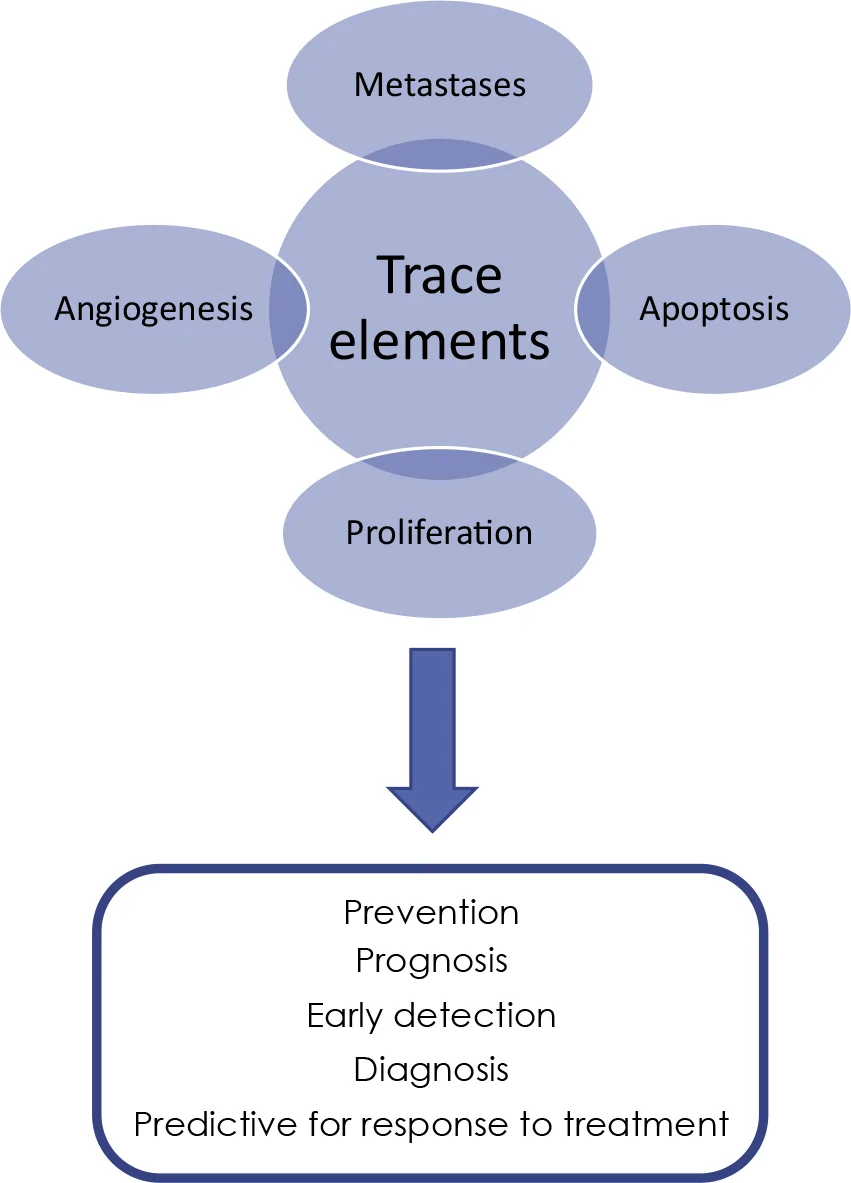
Potential roles of trace elements in oncology

Recent findings highlight the potential of trace element identification as a cancer biomarker.^17^ Imbalances in the Cu/Zn ratio may be used for clinical diagnosis and as a predictive biomarker for treatment response. Cp correlates with immune infiltration and serves as a prognostic biomarker in breast cancer.^17^ Elevated serum Cu-Cp levels have been found in lung, colon, ovarian, and bile duct cancers, while Cp expression is down-regulated in adrenocortical and hepatocellular carcinoma.^17^ Serum Cu levels increase in several cancers. In hepatocellular carcinoma, blood Cu and sulphur (S) are enriched in light isotopes compared to healthy individuals, and isotopic ratios of Cu (^65^Cu/^65^Cu) and S (^32^S/^32^S) may serve as disease biomarkers.^19^ Changes in the isotopic compositions of Fe, Cu, and Zn and their plasma concentrations in haematological malignancies can be measured for prognostic assessment.^20^ Further investigation is needed to fully evaluate the biomarker potential of trace metal concentrations, speciation, and isotopic fractionation. The potential roles of trace elements in oncology are shown in Figure 1.

## Trace elements as biomarkers of gastrointestinal (GI) cancers in clinical trials

Much research has focused on the role of trace elements in biochemical and physiological processes and their involvement in tumour growth, invasion, and metastasis. However, there are few published clinical studies examining the role of trace elements as prognostic and predictive biomarkers in GI cancer patients. Key clinical studies are summarized in Table 1.

**TABLE 1.** Overview of the data on the clinical impact of trace elements in gastrointestinal (GI) cancers

Author, year of publication, Ref	GI cancer type	Clinical study	Trace elements and analytical methods	Summary of main findings
Lossow *et al*., 2021^21^	Colorectal	Retrospective	Se, Cu, Fe, Zn ICP-MS, SRXRF	Elevated Cu and low Zn serum concentrations in four cancer types (CRC, lung, prostate, breast) studied. Analysis of Cu and Zn could contribute to an early cancer diagnosis.
Yang *et al*., 2021,^22^	Liver, gastric, colorectal	Prospective, closed	Cu, Zn, Fe, Se ICP-MS	OXs exists in the occurrence and development of cancer, related to the changes of trace element concentrations.
Stepien *et al*., 2017^23^	Colorectal	Prospective, closed	Cu, Zn XRF	Cu/Zn ratio may be associated with increased CRC risk, particularly within two years of diagnosis, and could serve as an early indicator of CRC development. Zn showed a potential protective effect, especially in women.
Baszuk *et al*., 2021^24^	Colorectal	Retrospective	Cu ICP-MS	A high blood Cu level (>900 μg/L) is associated with a significantly increased risk of colorectal cancer in the Polish population. Cu concentrations has the potential as a marker for identifying of patients for further surveillance with colonoscopy.
Nawi *et al*., 2019^25^	Colorectal	Retrospective	Ca, Cu, Mg, Mn, Se, Si, Zn, Co, S, Cd, Cr, Cu, Mg, Mn, Pb AAS	Serum concentrations of Ca, Cu, Mg, Mn, Se, Si, and Zn were lower in CRC patients, whereas Co and S the levels were higher. Concentrations of Cd, Cr, Cu, Mg, Mn, Pb and Zn were elevated in patients with metastasis.
Lener *et al*., 2016^26^	Pancreatic cancer	Prospective, closed	Se, Cu ICP-MS	Low Se and elevated Cu levels may contribute to PC development, higher Se concentrations are associated with longer survival in affected patients.
Yanjun *et al*., 2024^27^	Pancreatic cancer	Prospective, closed	Cu, Fe, Zn, Mn ICP-MS	High concentration levels of Cu may increase the risk of PC. Fe can promote ferroptosis, Excessive Fe levels may elevate PC risk. High Zn intake is associated with a reduced risk of PC and can inhibit tumour growth. Mn contributes to anti-PC effects primarily by promoting ferroptosis and suppressing excessive cell proliferation.
Türkdoğan *et al*., 2022^28^	Oesophageal, gastric, colorectal	Prospective, closed	Cd, Co, Cu, Fe, Mg, Mn, Pb, Zn, Ni FAAS	Cd, Co, Ni, Fe, and Mn were significantly lower in cancer patients compared to healthy controls. Serum Zn levels were lower in cancer patients, the difference was not statistically significant. No significant differences were observed for Cu, Mg, Pb, and Zn between cancer patients and controls.
Kocak *et al*., 2025^29^	Oesophageal squamous cell carcinoma	Prospective, closed	Al, Cr, Mn, Fe, Co, Cu, Zn, Se, Sb, Hg Pb ICP-MS	Significant increases in Cu, and Fe levels, as well as total oxidant status, alongside a marked decrease in Se levels in cancerous tissues.
Yan *et al*., 202^43^0	Hepatocellular cancer, gastric cancer	Prospective, closed	As, Cd, Co, Cr, Cu, Fe, Mn, Ni, Pb, Se, Zn ICP-MS	Tissue concentrations of As, Cd, Co, Cr, Cu, Fe, Mn, Ni, Pb, Se, and Zn in patients with liver cancer were significantly lower than those in healthy controls. Patients with gastric cancer exhibited lower levels of Cd, Co, Cr, Mn, Ni, and Zn, but higher levels of Cu and Se compared to the controls. Patients with liver and gastric cancers who had poorly differentiated tumours and positive lymph node metastases showed lower levels of trace elements.
Gupta *et al*., 2005^31^	Gallbladder cancer	Prospective, closed	Cu, Zn FAAS	Mean serum Zn levels, biliary and tissue Zn levels were significantly lower in gallbladder carcinoma patients comparing to patients with cholelithiasis, and healthy controls. Mean serum Cu levels, biliary and tissue Cu levels were significantly higher in gallbladder carcinoma patients comparing to those with cholelithiasis, and healthy controls. Serum Cu/Zn ratio showed a gradual and significant increase, rising from healthy controls to patients with cholelithiasis and patients with carcinoma of the gallbladder. Biliary and tissue Cu/Zn ratios were significantly higher in gallbladder carcinoma patients than in patients with cholelithiasis.
Basu *et al*., 2013^32^	Biliary tract cancers	Prospective, closed	Se, Zn, Cu, Mg, Cd, Cr, Pb, Ni FAAS	Se and Zn levels were significantly reduced, and Cu levels were significantly higher in serum, bile, and gallbladder tissue from gallbladder carcinoma patients. Pb, Cd, Cr and Ni levels were increased in serum and bile of these patients.
Stepien *et al*., 2017^33^	Hepatocellular cancer, biliary tract cancers	Prospective, closed	Cu, Zn XRF	Zn may play a role in preventing liver cancer development. An inverse association between pre-diagnostic Zn levels, but not Cu levels, and the risk of HCC is shown. An imbalance of Cu relative to Zn, indicated by a higher Cu/Zn ratio, was positively associated with HCC risk.
Reberšek *et al*., 2024^34^	Biliary tract cancers	Prospective, ongoing	Cu, Zn, Fe, se, Mn ICP-MS	Serum levels of trace elements, their proportion of free Cu and Cu-Cp, and its isotopic fractionation (Cu^65^/Cu^63^) are being investigated as potential predictive biomarkers of response to systemic therapy.
Kozlica *et al*., 2025^35^	Biliary tract cancers	Retrospective - prospective	Cu, Cu-Cp ICP-MS	Analytical methodologies for studying metabolic disorders affecting Cu metabolism. Accurate interpretation of disease states related to Cu disorders and detailed information obtained through advanced analytical techniques. Immunological assays, ultrafiltration procedures, and speciation techniques based on ICP-MS including Cu isotopic analysis for identification of metabolic abnormalities in different diseases and types of cancer, including BTCs.

Al = aluminium; As = arsenic, AAS = atomic absorption spectrometry; BTCs = biliary tract cancers; Ca = calcium; Cd = cadmium; Cr = chromium; Co = cobalt; CRC = colorectal cancer; Cu-Cp = copper bound ceruloplasmin; Cu = copper; FAAS = flame atomic absorption spectrometry; HCC = hepatocellular cancer; Hg = mercury; ICP-MS = inductively coupled plasma mass spectrometry; Fe = iron-Fe; Mg = magnesium; Mn = manganese; Ni = nickel; OxS = oxidative stress; Pb = lead; PC = pancreatic cancer; Sb = antimuon; SE = selenium; Si = silicon; SRXRF = synchrotron radiation based X-ray fluorescence; XRF = X-ray fluorescence; Zn = zinc-Zn

## Conclusions and future directions

Predictive and prognostic biomarkers are essential for personalized medicine and optimal treatment of cancer patients. Trace elements as biomarkers in oncology represent a promising field for the detection, diagnosis, and prediction of treatment response. Currently, serum determination of trace elements as prognostic or predictive biomarkers has not been integrated into routine clinical practice. Few clinical studies have examined the role of trace element concentrations in predicting prognosis, survival, and treatment response in GI cancer patients or their potential as therapeutic targets.

Trace elements such as Cu, Zn, and Fe, including exchangeable and Cp-bound copper and isotope ratios in serum, are emerging as promising biomarkers for prognostic and predictive purposes in systemic cancer treatment. However, the evidence remains inconsistent and varies by cancer type. Future research should focus on developing accurate, reliable, and optimized analytical and imaging methods for the quantitative determination of serum trace elements and investigating their role in cancer diagnosis and treatment. More clinical research is needed to define the significance of trace elements in relation to prognosis, cancer characteristics, disease stage, and treatment outcomes.

## References

[1] World Health Organization International Agency for Research on Cancer (IARC) GLOBOCAN 2020: Estimated cancer incidence, mortality and prevalence worldwide in 2020.

[2] Cancer in Slovenia 2022 (2025).

[3] National Comprehensive Cancer Network: NCCN Clinical Practice Guidelines in Oncology: Colon cancer. V 2 (2025). https://www.nccn.org/professionals/physician_gls/pdf/colon.pdf.

[4] Cervantes A, Adam R, Roselló S, Arnold D, Normanno N, Taïeb J (2023). ESMO Guidelines Committee. Metastatic colorectal cancer: ESMO Clinical Practice Guideline for diagnosis, treatment and follow-up. Ann Oncol.

[5] Lowe RC, Anderson CD. Clinical presentation, diagnosis, and staging of cholangiocarcinoma. UpToDate.

[6] Ferreira CR, Gahl WA. (2017). Disorders of metal metabolism. Transl Sci Rare Dis.

[7] Morales M, Xue X. (2021). Targeting iron metabolism in cancer therapy. Theranostics.

[8] Stojsavljević A, Vujotić L, Rovčanin B, Borković-Mitić S, Gavrović-Jankulović, Manojlović D. (2020). Assessment of trace metal alterations in the blood, cerebrospinal fluid and tissue samples of patients with malignant brain tumors. Sci Rep.

[9] Lelièvre P, Sancey L, Coll JL, Deniaud A, Busser B. (2020). The multifaceted roles of copper in cancer: a trace metal element with dysregulated metabolism, but also a target or a bullet for therapy. Cancers.

[10] Chen F, Han B, Meng Y, Han Y, Liu B, Zhang B (2021). Ceruloplasmin correlates with immune infiltration and serves as a prognostic biomarker in breast cancer. Aging (Albany NY).

[11] Wang B, Wang XP. (2019). Does ceruloplasmin defend against neurodegenerative diseases?. Curr Neuropharmacol.

[12] Linder MC. (2016). Ceruloplasmin and other copper binding components of blood plasma and their functions: an update. Metallomics.

[13] Mukae Y, Ito H, Miyata Y, Araki K, Matsuda T, Aibara N (2021). Ceruloplasmin levels in cancer tissues and urine are significant biomarkers of pathological features and outcome in bladder cancer. Anticancer Res.

[14] Sogabe M, Kojima S, Kaya T, Tomioka A, Kaji H, Sato T (2022). Sensitive new assay system for serum wisteria floribunda agglutinin-reactive ceruloplasmin that distinguishes ovarian clear cell carcinoma from endometrioma. Anal Chem.

[15] Michalczyk K, Cymbaluk-Płoska A. (2020). The Role of zinc and copper in gynecological malignancies. Nutrients.

[16] Lossow K, Schwarz M, Kipp AP. (2021). Are trace element concentrations suitable biomarkers for the diagnosis of cancer?. Redox Biol.

[17] Chen F, Han B, Meng Y, Han Y, Liu B, Zhang B (2021). Ceruloplasmin correlates with immune infiltration and serves as a prognostic biomarker in breast cancer. Aging (Albany NY).

[18] Bolann BJ, Rahil-Khazen R, Henriksen H, Isrenn R, Ulvik RJ. (2007). Evaluation of methods for trace-element determination with emphasis on their usability in the clinical routine laboratory. Scand J Clin Lab Invest.

[19] Balter V, Nogueira da Costa A, Bondanese VP, Jaouen K, Lamboux A (2015). Natural variations of copper and sulphur stable isotopes in blood of hepatocellular carcinoma patients. Proc Natl Acad Sci U S A.

[20] Hastuti AAMB, Costas-Rodríguez M, Matsunaga A, Ichinose T, Hagiwara S, Shimura M (2020). Cu and Zn isotope ratio variations in plasma for survival prediction in hematological malignancy cases. Sci Rep.

[21] Lossow K, Schwarz M, Kipp AP. (2021). Are trace element concentrations suitable biomarkers for the diagnosis of cancer?. Redox Biol.

[22] Yang YW, Dai CM, Chen XH, Feng JF. (2021). The relationship between serum trace elements and oxidative stress of patients with different types of cancer. Oxid Med Cell Longev.

[23] Stepien M, Jenab M, Freisling H, Becker NP, Czuban M, Tjønneland A (2017). Prediagnostic copper and zinc biomarkers and colorectal cancer risk in the European Prospective Investigation into Cancer and Nutrition cohort. Carcinogenesis.

[24] Baszuk P, Marciniak W, Derkacz R, Jakubowska A, Cybulski C, Gronwald J (2021). Blood copper levels and the occurrence of colorectal cancer in poland. Biomedicines.

[25] Nawi AM, Chin SF, Azhar Shah S, Jamal R. (2019). Tissue and serum trace elements concentration among colorectal patients: a systematic review of case-control studies. Iran J Public Health.

[26] Lener MR, Scott RJ, Wiechowska-Kozłowska A, Serrano-Fernández P, Baszuk P, Jaworska-Bieniek K (2016). Serum concentrations of selenium and copper in patients diagnosed with pancreatic cancer. Cancer Res Treat.

[27] Yanjun Y, Jing Z, Yifei S, Gangzhao G, Chenxin Y, Qiang W (2024). Trace elements in pancreatic cancer. Cancer Med.

[28] Türkdoğan MK, Karapinar HS, Kilicel F. (2022). Serum trace element levels of gastrointestinal cancer patients in an endemic upper gastrointestinal cancer region. J Trace Elem Med Biol.

[29] Kocak OF, Yaman ME, Eroglu A. (2025). An integrated analytical approach for biomarker discovery in esophageal cancer: combining trace element and oxidative stress profiling with machine learning. J Trace Elem Med Biol.

[30] Yan J, Zhang H, Zhang M, Tian M, Nie G, Xie D (2024). The association between trace metals in both cancerous and noncancerous tissues with the risk of liver and gastric cancer progression in northwest China. J Pharm Biomed Anal.

[31] Gupta SK, Singh SP, Shukla VK. (2005). Copper, zinc, and Cu/Zn ratio in carcinoma of the gallbladder. J Surg Oncol.

[32] Basu S, Singh MK, Singh TB, Bhartiya SK, Singh SP, Shukla VK. (2013). Heavy and trace metals in carcinoma of the gallbladder. World J Surg.

[33] Stepien M, Hughes DJ, Hybsier S, Bamia C, Tjønneland A, Overvad K (2017). Circulating copper and zinc levels and risk of hepatobiliary cancers in Europeans. Br J Cancer.

[34] Rebersek M, Hribernik N, Markovic K, Markovic S, Valentinuzzi KU, Cemazar M (2024). Determination of copper and other trace elements in serum samples from patients with biliary tract cancers: prospective noninterventional nonrandomized clinical study protocol. Radiol Oncol.

[35] Kozlica K, Milačič Ščančar R, Reberšek M, Čemažar M, Uršič Valenzinuzzi K, Ščančar J. (2025). Advances in copper speciation, isotopic ratio measurement, bioimaging, and single-cell analysis – a critical review. TrAC trends in analytical chemistry.

